# Clinical characteristics of treatment-resistant depression in adults in Hungary: Real-world evidence from a 7-year-longz retrospective data analysis

**DOI:** 10.1192/j.eurpsy.2022.141

**Published:** 2022-09-01

**Authors:** Z. Rihmer

**Affiliations:** Semmelweis University, Department Of Psychiatry And Psychotherapy, Budapest, Hungary

**Keywords:** Antidepressants, Hungary, Treatmen-resistant depression, Mortality

## Abstract

Treatment-resistant depression (TRD) is associated with poor quality of life, elevated morbidity and mortality and high economic burden.Our observational retrospective epidemiological study have estimated the rate of patients with TRD within a cohort of major depressive disorder (MDD) patients in Hungary and examine the comorbidities and mortality of patients with and without TRD.Our study included patients with MDD who experienced new onset of depressive episode and received antidepressant prescription between 01 January 2009 and 31 August 2015, using data from nationwide, longitudinal database. A patient was considered to have TRD if two different antidepressant treatments had failed during a given pharmacologically treated periode.Overall, 99,531 MDD patients were included, of which 8,268 (8,3%) met the criteria of TRD. Patients with TRD had significantly higher rate of having “Neurotic, stress-related and somatoform disorders”, autoimmune disorders, cardio-or cerebrovascular diseases, thyroid disorders and suicide attempts than non-TRD patients (for all comparisons, p < 0,005).Ths first study to assess the frequency of TRD in Hungary have found that the proportion of TRD is in the same range as in studies with similar methodology reported form other countries. The majority of our other main findings are also in line with previous studies from other countries.

**Disclosure:**

This study was founded by Janssen Pharmaceutical Companies of Johnsson and Johnsson. I, as scientific advisor/consultant have received honoraria as I have participated in conceptualization, investigation, validation and writing this lecture.

